# Draft Genome Sequences of 16 Halophilic Prokaryotes Isolated from Diverse Environments

**DOI:** 10.1128/MRA.01540-19

**Published:** 2020-02-20

**Authors:** Joel Rodriguez-Medina, Hyunsoo G. Kim, Julia Castro, Cameron M. Contreras, Celine L. Glon, Aditi Goyal, Bonnie Y. Guo, Sarai Knowles, Jason C. Lin, Casey L. McGuiness, Eldar Sorkin, Jordan Stefani, Sai J. Yegireddi, Shyama Chaganti, Dante Cui, Samuel L. Deck, Yashvi Deokule, Hallie Douglas, Matthew Kenaston, Alana O’Brien, Emily Patterson, Nathan Schoppa, Dean Tran Vo, Kelly Tran, Thuy-Linh Tran, Valeria Pérez-Irizarry, Krismarie Carrasquillo-Nieves, Rafael Montalvo-Rodriguez, Andrew I. Yao, John G. Albeck, Marc T. Facciotti, Alex S. Nord, Robert E. Furrow

**Affiliations:** a University of California, Davis, Davis, California, USA; b University of Puerto Rico, Mayagüez, Mayagüez, Puerto Rico; Portland State University

## Abstract

Halophile-specific enzymes have wide-ranging industrial and commercial applications. Despite their importance, there is a paucity of available halophile whole-genome sequences. Here, we report the draft genome sequences of 16 diverse salt-tolerant strains of bacteria and archaea isolated from a variety of high-salt environments.

## ANNOUNCEMENT

Halophiles have the ability to survive and thrive in high salinity. Halophile-specific enzymes can be harnessed for use in a variety of commercial and industrial applications, making them a valuable target for study (1, 2). As part of a two-quarter course series, undergraduate students isolated and cultured microbes obtained from several high-salinity substrates (Table 1). All samples from Puerto Rico were from salt flats in Cabo Rojo. Halobacterium medium 372 was used as the culturing medium (https://www.dsmz.de/microorganisms/medium/pdf/DSMZ_Medium372.pdf). To isolate a wider variety of strains, samples were plated in medium 372 at multiple salinity levels, either 100, 150, 200, or 250 g NaCl per 1,000 ml of medium. To grow a diversity of strains, we used both direct-plated samples and samples plated from liquid enrichment cultures. For solid samples, direct plating required hydrating the sample with H_2_O at a density of 200 g of sample per liter. To create enrichment cultures, we combined 100 μl of sample with 3 ml of liquid medium and incubated it at 37°C until turbid in ≤7 days. Colonies were grown by spread plating of the prepared samples. Pure liquid cultures were created from isolated plate colonies and grown at 37°C until turbid in ≤7 days. Purity was assessed using microscopy and visual analysis of streak-plated pure cultures.

**TABLE 1:** Summary of the draft genome sequences for 16 microbes isolated from high-salt environments and culinary salt

Species (% identity)	Isolate source (medium salinity [g/liter NaCl])	No. of reads	Median insert size (bp)	No. of contigs of ≥0 bp	Total length (bp)	G+C %	*L*_50_	*N*_50_ (bp)
Bacillus hwajinpoensis (99.79)	French fleur de sel salt from grocery store (100)	560,183	433	87	4,387,231	40.22	3	797,473
Halobacillus halophilus (98.21)	Solar salt harvested from saltern, Puerto Rico (100)	505,256	448	51	3,841,848	47.38	3	575,568
Halobacillus litoralis (99.23)	Sand harvested near saltern crystallizers, Puerto Rico (100)	1,228,604	197	258	4,087,490	43.59	2	1,038,528
*Halobacillus litoralis* (99.36)	Sand harvested near saltern crystallizers, Puerto Rico (100)	1,488,864	215	92	3,984,713	43.83	3	396,878
*Halobacillus litoralis* (99.04)	Filtered 34% NaCl saltern water, Puerto Rico (100)	495,194	484	65	3,850,954	47.23	5	237,386
*Halobacillus litoralis* (99.1)	Filtered 34% NaCl saltern water, Puerto Rico (100)	1,866,405	211	577	4,062,334	47.67	2	690,789
Pontibacillus yanchengensis (99.39)	French fleur de sel salt from grocery store (200)	454,979	275	75	4,476,898	38.35	7	200,610
*Pontibacillus yanchengensis* (99.39)	Filtered 34% NaCl saltern water, Puerto Rico (200)	1,370,382	221	93	4,441,339	38.43	3	575,184
Virgibacillus halodenitrificans (100)	Sand harvested near saltern crystallizers, Puerto Rico (100)	1,634,098	238	2,151	5,319,423	37.39	155	6,747
*Virgibacillus halodenitrificans* (99.87)	Solar salt harvested from saltern, Puerto Rico (100)	399,182	482	109	4,012,173	37.36	6	215,037
Virgibacillus massiliensis (99.74)	Sand harvested near saltern crystallizers, Puerto Rico (100)	1,323,006	239	166	4,260,595	36.96	2	1,149,539
Halomonas alkaliantarctica (97.5)	Filtered 34% NaCl saltern water, Puerto Rico (200)	1,230,594	293	91	3,324,735	58.54	4	316,952
Halomonas utahensis (99.87)	French fleur de sel salt from grocery store (100)	287,001	378	57	3,351,671	62.12	6	219,081
*Halomonas utahensis* (99.87)	French fleur de sel salt from grocery store (150)	2,659,128	227	64	3,361,279	62.12	4	422,380
Halorubrum terrestre (100)	Solar salt harvested from saltern, Puerto Rico (250)	999,388	393	90	3,385,208	67.81	11	97,966
*Halorubrum terrestre* (99.86)	Solar salt harvested from saltern, Puerto Rico (150)	1,461,031	315	885	3,678,879	65.29	9	146,067

Genomic DNA was isolated from the liquid cultures using a Wizard genomic DNA purification kit (Promega, Madison, WI, USA). MicrobesNG (Birmingham, UK) performed library preparation, sequencing, adapter trimming, and assembly (full protocol available at https://microbesng.com/documents/5/MicrobesNG_Methods_Document_-_PDF.pdf). DNA was quantified using the Quant-iT double-stranded DNA (dsDNA) high-sensitivity (HS) assay (Thermo Fisher Scientific, Waltham, MA, USA). Libraries were prepared using a Nextera XT library prep kit (Illumina, San Diego, CA, USA) with the following two protocol modifications: using 2 ng of DNA instead of 1 ng and adjusting the PCR elongation time to 1 minute. Libraries were sequenced using an Illumina HiSeq instrument with a 250-bp paired-end protocol. A mean of 1,122,706 reads per strain were generated by the sequencing. Reads were trimmed using Trimmomatic 0.30 with a sliding window quality cutoff of Q15 (3). SPAdes 3.7 (4) was used for *de novo* assembly with recommended settings for 250-bp paired-end reads. The mean coverage ranged from 35 to 289×. The mean *N*_50_ value was 442,887 bp with a mean *L*_50_ of 14.1.

The taxonomy was estimated with BLAST (5) using the 16S rRNA gene sequences of each strain. We identified 14 bacteria (genera *Halobacillus*, *Pontibacillus*, *Virgibacillus*, *Bacillus*, and *Halomonas*) and 2 archaea (genus *Halorubrum*). The top query results all had above a 97% identity (99.4% mean identity).

### Data availability.

All 16 whole-genome sequences and SRA files have been deposited at DDBJ/ENA/GenBank as a BioProject under accession number PRJNA587497. A 17th sample is also included in the BioSample data for the BioProject, but this sample appeared to be a mix of two species and could not be assembled into a high-quality genome.
