# Correction for Rodriguez-Medina et al., “Draft Genome Sequences of 16 Halophilic Prokaryotes Isolated from Diverse Environments”

**DOI:** 10.1128/MRA.00239-20

**Published:** 2020-04-09

**Authors:** Joel Rodriguez-Medina, Hyunsoo G. Kim, Julia Castro, Cameron M. Contreras, Celine L. Glon, Aditi Goyal, Bonnie Y. Guo, Sarai Knowles, Jason C. Lin, Casey L. McGuiness, Eldar Sorkin, Jordan Stefani, Sai J. Yegireddi, Shyama Chaganti, Dante Cui, Samuel L. Deck, Yashvi Deokule, Hallie Douglas, Matthew Kenaston, Alana O’Brien, Emily Patterson, Nathan Schoppa, Dean Tran Vo, Kelly Tran, Thuy-Linh Tran, Valeria Pérez-Irizarry, Krismarie Carrasquillo-Nieves, Rafael Montalvo-Rodriguez, Andrew I. Yao, John G. Albeck, Marc T. Facciotti, Alex S. Nord, Robert E. Furrow

**Affiliations:** a University of California, Davis, Davis, California, USA; b University of Puerto Rico, Mayagüez, Mayagüez, Puerto Rico

## ANNOUNCEMENT

Volume 9, no. 8, e01540-19, 2020, https://doi.org/10.1128/MRA.01540-19. Page 2: Lines 3 and 4 of Acknowledgments should read as follows. “This work was supported by a Howard Hughes Medical Institute Professor grant awarded to Mark Goldman (grant number 52008137).

We thank Jonathan Eisen, Katherine Dahlhausen, Mark Goldman, Ashley Vater, and David Coil for help with course design and planning.”

